# Thy-1 in Integrin Mediated Mechanotransduction

**DOI:** 10.3389/fcell.2019.00022

**Published:** 2019-02-25

**Authors:** Ping Hu, Thomas H. Barker

**Affiliations:** Department of Biomedical Engineering, University of Virginia, Charlottesville, VA, United States

**Keywords:** Thy-1, integrin, *trans* interaction, *cis* interaction, mechanotransduction

## Abstract

The glycosylphosphatidylinositol (GPI) anchored glycoprotein Thy-1 has been prevalently expressed on the surface of various cell types. The biological function of Thy-1 ranges from T cell activation, cell adhesion, neurite growth, differentiation, metastasis and fibrogenesis and has been extensively reviewed elsewhere. However, current discoveries implicate Thy-1 also functions as a key mechanotransduction mediator. In this review, we will be focusing on the role of Thy-1 in translating extracellular mechanic cues into intracellular biological cascades. The mechanotransduction capability of Thy-1 relies on *trans* and *cis* interaction between Thy-1 and RGD-binding integrins; and will be discussed in depth in the review.

As the smallest member of the immunoglobulin superfamily, Thy-1 (CD90) is a 25–37 kDa glycosyl phosphatidylinositol (GPI) anchored cell membrane protein that bears critical biological functions. The glycoprotein is expressed across many different cell types including fibroblasts, endothelial cells, neuron and hematopoietic cells (Craig et al., 1993; Rege and Hagood, 2006b). Since its discovery decades ago, extensive scrutiny on the glycoprotein has established Thy-1 as an important player in almost every aspect in cellular biology including adhesion, migration, apoptosis, wound healing, tumorigenesis and fibrogenesis (Barker et al., 2004; Sanders et al., 2007, 2008; Barker and Hagood, 2009; Lee et al., 2013). More recently, studies have connected Thy-1 with mechanotransduction, specifically through its interaction with integrins. This mini review will focus on the role of Thy-1 in integrin mediated mechanotransduction, with a broader scope on Thy-1 driven physiological responses via mediating conversion of extracellular biophysical cues into intracellular biochemical signals.

## Thy-1-integrin Interaction, *cis* and *trans*

Integrins are a group of adhesion receptors connecting the extracellular matrix (ECM) with the cell cytoskeleton through their bulky, dimeric head domain, a type I transmembrane domain and a relatively small cytoplasmic domain (Luo et al., 2007). Integrins have long been regarded as critical mechanotransducers since the direct engagement between integrins and their ECM ligands is the prerequisite of formation of focal adhesions and cellular contractility. The evidence of Thy-1-integrin interaction began to emerge in the past decade. Potential interactions between Thy-1 and integrin αvβ5 has been proposed as the mechanism of Thy-1 mediated signaling that blocks activation of TGF-β (Herrera-Molina et al., 2013). Similarly, Thy-1 positive lung fibroblasts are resistant to TGF-β activation induced lung fibrosis, implicating possible role of Thy-1 in suppressing αvβ6 mediated TGF-β activation (Zhou et al., 2004). Direct interactions have been shown between Thy-1 and integrin αvβ3 on astrocytes (Leyton et al., 2001). The interaction is mediated through the RLD motif on the recombinant Thy-1-FC molecule and the engagement between Thy-1 and αvβ3 can promote focal adhesion formation as well as FAK phosphorylation. Another study later discovered that this *trans* interaction between Thy-1 and integrin αvβ3 induces Thy-1 microclustering and colocalization with Csk-binding protein (CBP) while displacing Src kinase from these clusters at the same time (Maldonado et al., 2017). Melanoma cells have also been seen to exploit Thy-1 expressed by vascular endothelial cells for adhesion and subsequent tumor metastasis, presumably through Thy-1- αvβ3 interaction (Schubert et al., 2013). αvβ3 is not the only integrin that has shown capability to interact with Thy-1. Thy-1-α5β1 and syndecan4 can form triplex and behave as a catch bond (Fiore et al., 2014).

While *trans* interactions between Thy-1 and integrin apparently mediates mechanotransduction, little is known regarding the impact of *cis* interaction until lately. In a study published by Fiore and his colleagues, Thy-1 is found to interact with integrin αvβ3 in *cis* on the surface of lung fibroblasts (Fiore et al., 2015). The interaction helps to keep the integrin in a low affinity, bent conformation. Moreover, the interaction facilitates Fyn, a member of SFK critical in mechanosignaling, recruitment to focal adhesions while also keeps c-Src activity under check through recruitment of CBP.

## *Trans* Interaction Between Thy-1 and Integrin αvβ3 Mediates Mechanotransduction

Thy-1 has been shown to support cell adhesion through *trans* interaction with integrin. Immobilized Thy-1 is capable to function as ligand for integrin αvβ3 and support cell adhesion in a Mn^2+^ dependent manner. On the cell membrane, interactions between αvβ3 on DITNC1 astrocytes and Thy-1 on neuron cells support cell adhesion but inhibit neuron cell differentiation and neurite extension (Herrera-Molina et al., 2012). Immobilized recombinant αvβ3-FC functions similarly and induces clustering of Thy-1 on neuron cell surface. It has been proposed that such a *trans* interaction triggered redistribution/clustering of Thy-1 leads to inactivation of Src through Thy-1 mediated CBP recruitment. Thy-1 mediated cell-cell interaction has also been found to be critical for melanoma cell adhesion and metastasis. Thy-1 deficient mice showed significantly reduced metastasis sites due to ablation of Thy-1 mediated melanoma cell adhesion on Endothelial cells (Schubert et al., 2013). When mediating cell-cell adhesion, Thy-1 not only needs to interact with integrin αvβ3, but also need to bring in Syndecan4, a lipid raft protein that binds to a heparin-binding domain on Thy-1. The interaction between Thy-1 and Syndecan4 itself is not sufficient to induce Rac-1 RhoGTPase activation; however, the binding is required for the Thy-1- αvβ3 interaction to support cell adhesion and migration (Kong et al., 2013). It's worth noting that the Thy-1- αvβ3 interaction alone indeed triggers phosphorylation of Akt, indicating that cell-cell *trans* interaction through Thy-1 and integrin could promote cell viability/survival but is not sufficient to generate mechano-signal transduction. Interestingly, while surface Thy-1 clustering induced by integrin αvβ3 generates inhibitory signal in neuron cells, Thy-1 crosslinking by mAb induces Ca^2+^ influx and proliferation in T lymphocytes (Kroczek et al., 1986; Conrad et al., 2009). The seemingly paradoxical evidence implicates highly context dependent nature of Thy-1 function.

Thy-1-FC conjugated beads are sufficient to induce enhanced formation of focal adhesions and elevated tyrosine phosphorylation of p130^cas^ and FAK (Leyton et al., 2001). A Thy-1-CBP-RhoA-ROCK axis has been proposed to induce astrocyte retraction and RhoA dependent actin stress fiber formation (Avalos et al., 2004; Maldonado et al., 2017). The phenomenon is induced via Thy-1-FC conjugated protein A beads, implicating that clustered Thy-1 is likely required to mediate the *trans*-interaction based mechanotransduction through integrin αvβ3. Unlike other traditional integrin ligands, Thy-1 is a monovalent molecule and its RLD motif likely binds with integrin at lower affinity. Therefore, clustering of Thy-1 and presence of potential binding partners in addition to integrin (e.g., syndecan4) could be essential for Thy-1 mediated cell-cell interaction and mechanotransduction. This is particularly plausible considering that immobilized Thy-1-FC can't support cell adhesion without the presence of Mn^2+^ whereas conjugated (and thus “clustered”) Thy-1-FC beads successfully induced focal adhesion assembly in a RhoA-ROCK dependent pathway (Leyton et al., 2001; Avalos et al., 2004). Interaction with ECM ligands induces integrin clustering which is the key event in cell adhesion and migration. It is known that integrin clustering is dependent on PI(4,5,)P_2_ and Talin (Cluzel et al., 2005; Saltel et al., 2009) while syndecan4 helps retention of PI(4,5,)P_2_ in cell membrane (Kwon et al., 2009). Therefore, Thy-1, integrin αvβ3 and syndecan4 work synergistically to mediate mechanotransduction through cell-cell interaction. Further downstream, this trimolecular complex also regulates RhoA GTPase mainly through modulating p^190^GAP phosphorylation and distribution. Syndecan4 and integrin α5β1 have been shown to regulate p^190^GAP membrane distribution and Src-dependent tyrosine phosphorylation, respectively (Bass et al., 2008). The coordinated interaction subsequently leads to suppressed RhoA activity and cell migration. However, introduction of Thy-1 causes a reduction of Src activity and downregulation of p^190^GAP, which leads to higher RhoA activity, stable adhesion and enhanced stress fiber formation (Barker et al., 2004). The phenomenon can be attributed, in part, to the recruitment of CBP by Thy-1 to integrin membrane proximity, which leads to inhibitory phosphorylation of Src kinase by CBP interacting Csk. More interestingly, the Thy-1- α5β1-Syndecan4 trimolecular complex not only delivers mechano-related biochemical signaling coordinately but also physically interprets force directly (Fiore et al., 2014). When Thy-1 binds to either α5β1 integrin or Syndecan4 alone, both interactions behave as classic slip bond, meaning that the lifetime of the interactions decreases with force application. However, the trimolecular bond expresses a unique catch bond feature–described as “dynamic catch” by the authors. The mechanism behind the phenomenal has been proposed as a sudden bond stiffening from an acquired contribution of the syndecan4-Thy-1 interaction, once the force load reaches a ~15 pN threshold. Before reaching the threshold, α5β1-Thy-1 interaction bears the majority of the force whereas after the threshold, due to force-induced extension of the GAG motif on Syndecan4, both α5β1 and Syndecan4 start to resist force at full load. Taken together, the Thy-1-α5β1-Syndecan4 complex mediates mechanotransduction both at the single molecule biophysical level and at the cell biochemical level.

## Mechanotransduction in *cis*

In contrast to only the induction of focal adhesions and promotion of FAK activation seen in *trans, cis* interaction between Thy-1 and integrin is more complicated, providing both a tonic inhibition, but also facilitating efficient mechanosignaling in the focal adhesion (Fiore et al., 2015). The Thy-1- αvβ3 interaction shifts the dynamic equilibrium of integrin conformation toward a bent-closed state. This effectively reduces integrin avidity for its extracellular ligand; Thy-1 is a weak inhibitor of integrin in *cis*. Remarkably, the Thy-1-αvβ3 interaction physically couples unbound integrin to lipid raft microdomains containing critical signaling molecules. Thus, Thy-1 facilitates co-clustering of lipid raft proteins with focal adhesions enabling proper mechanosensing in fibroblasts. Integrin mediated mechanotransduction relies on ECM ligand engagement and subsequent integrin clustering, which leads to self-activation of FAK and Src, resulting in downstream RhoA activation and cellular contractility (Hu and Luo, 2013). More specifically, by keeping integrin in the bend-low affinity conformation, Thy-1 not only constraints the ligand accessibility for integrin but also limits the likelihood of ECM ligand binding independent self-clustering and thus reduces the overall integrin avidity. Thy-1 keeps c-Src activity in check through recruitment of the lipid raft protein CBP, which leads to recruitment of Src inhibitor Csk; concurrently the lipid raft-associated Src-family member Fyn is brought to the focal adhesion enabling a prompt mechanosignaling response after ligand engagement (Fiore et al., 2015). It is worth noting that this Thy-1-mediated mechanosensing requires proper lipid raft location, as replacing the GPI anchor with a CD8 transmembrane domain greatly reduced the ability of cells to appropriately respond to environmental rigidity. Lipid rafts have been widely regarded as a critical participant in mechanotransduction (Head et al., 2014). Colocalization of Fyn, CBP and another Thy-1 interacting protein Reggie1/2 on non-caveolar lipid raft has been reported previously (Stuermer et al., 2001; Deininger et al., 2003). Moreover, Fyn has been shown to be able to both interact with FAK in early integrin mediated adhesion and phosphorylate CBP, resulting in subsequent recruitment/activation of Csk (Yasuda et al., 2002; Maksumova et al., 2005; Baillat et al., 2008). Adding the evidence together, Thy-1 likely functions as a lipid raft coupler, recruiting Fyn and CBP to the focal adhesion to regulate basal Src activity through Csk. In addition to promote RhoA activity through downregulating c-Src dependent p^190^GAP activity, Fyn has also shown to directly phosphorylate and activate Rho guanine nucleotide exchange factor (GEF) in response to integrin mediated force transduction, resulting in a more direct activation of RhoA (Guilluy et al., 2011). Importantly, the activity of Rho GTPase is required for ECM stiffness induced nucleus translocation of Yap/Taz, which drives mechano-activation of fibroblast and fibrosis (Dupont et al., 2011; Liu et al., 2015). Taking together, the direct and indirect regulatory role of Fyn over RhoA activity makes it a core modulator of force-induced cellular response.

The third way Thy-1 appears to impact mechanotransduction is through regulating the TGF-β pathway. TGF-β-SMAD2/3/4 is well established as the main signaling route to induce mechano-related cellular responses including proliferation, cellular contraction and ECM deposition. The signaling axis is also the main driving force in fibrosis. It has been reported that Thy-1 null c57BL/6 mice were more prone to develop severe lung fibrosis after bleomycin treatment (Hagood et al., 2005). Thy-1 negative fibroblasts are more responsive toward inflammatory cytokines like TGF-β whereas Thy-1 positive cells are resistant to similar treatments. The difference does not appear to be due to downstream signal transduction of TGF-β but instead to higher latent TGF-β activation in Thy-1 negative cells (Zhou et al., 2004), potentially through Thy-1 stabilization of integrin's bent conformation as described above. Likewise, induction of MMP9 by TGF-β has been observed in Thy-1 negative fibroblasts but not in Thy-1 positive fibroblasts, implicating Thy-1 as an important suppressor in MMP9 induced latent TGF-β activation– the positive feedback loop that efficiently enhances TGF-β signaling (Ramirez et al., 2011). The interaction between Thy-1 and integrin αvβ5 has been proposed as a mechanism to constrain latent TGF-β activation by the integrin (Zhou et al., 2010). The study, however, failed to reveal if the inhibition is caused by *cis* interaction between the two molecules or *trans*. It is conceivable that by keeping TGF-β activating integrins (αvβ5 and αvβ6) in a low affinity conformation, Thy-1 can reduce activation of endogenous TGF-β, enabling a cellular “brake” to TGF-β.

## Conclusion

Thy-1 bears a vast range of functionality, affecting T cell activation, proliferation, differentiation, neuron regeneration, adhesion and fibrosis (Rege and Hagood, 2006a,b). Interestingly, many of these functions are overlapping with integrin functionalities such as immunological synapse formation (αLβ2; Springer and Dustin, 2012), proliferation, adhesion, etc. The dual integrin interacting pattern (*trans* and *cis*) makes Thy-1 a key mechanoregulator through its integrin interaction capacity. The subsequent biological impacts of Thy-1-integrin interactions can be further categorized as either on the plasmamembrane or in the cytosol.

**Figure 1:**
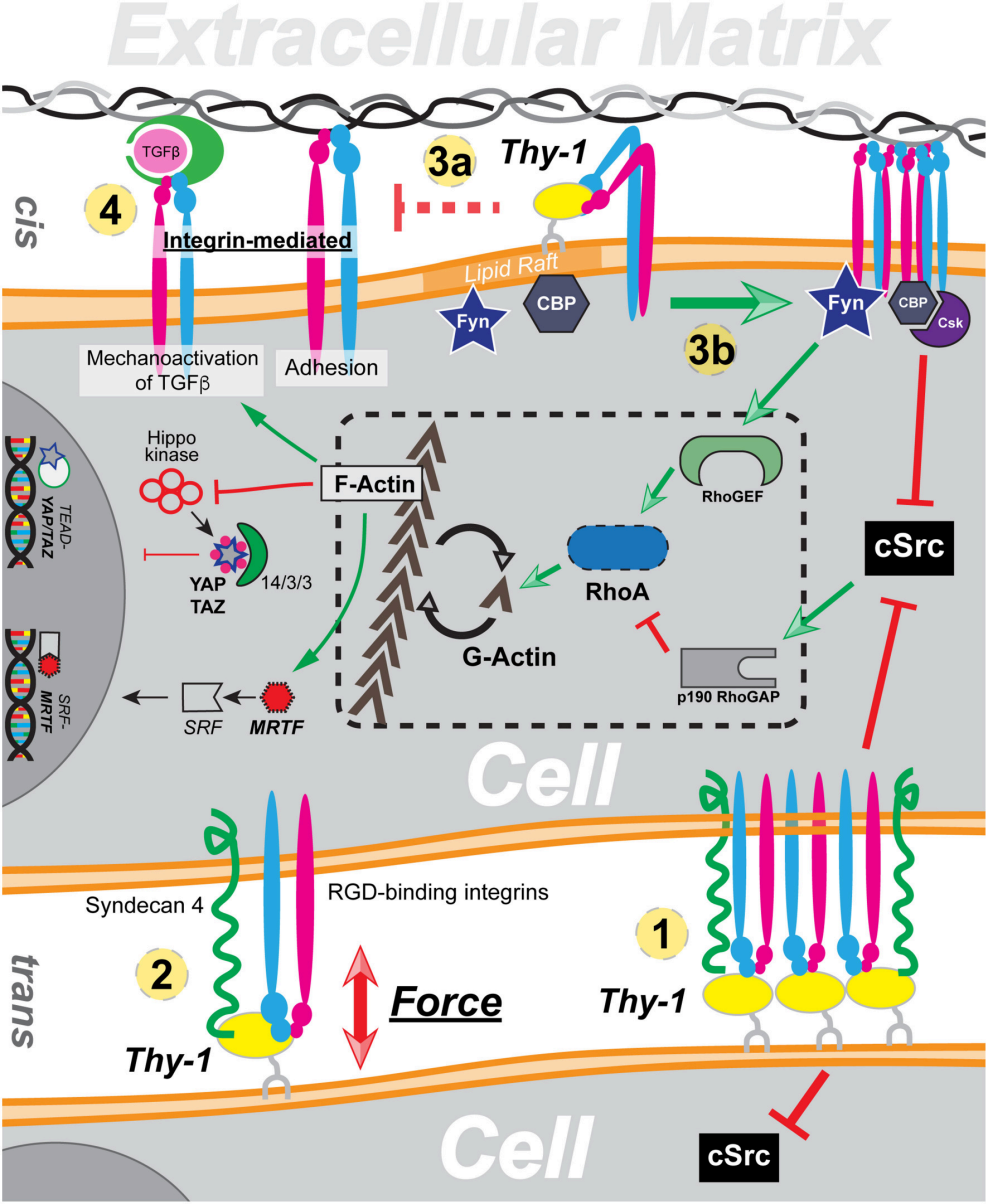
A global overview of Thy-1's functionality in mechanotransduction through cis and trans interaction with integrin. ① Trans interaction with integrin induces Thy-1 clustering and Src inhibition. ② Thy-1-integrin-Syndecan4 triplex directly responds to force by forming a dynamic catch bond. ③ Direct cis interaction between Thy-1 and integrin results in reduction of integrin affinity (a) and downstream regulation of integrin mediated mechanotransduction (b). ④ Thy-1-integrin cis interaction also suppresses integrin-dependent TGF-β activation.

On the plasma membrane, Thy-1 exerts profound impact in a direct manner. On the surface of neurons, Thy-1 directly binds to astrocyte integrin αvβ3 in *trans*. The interaction triggers Thy-1 clustering and suppresses neuron outgrowth (Leyton et al., 2001). Thy-1 dependent cell adhesion and migration is also mediated through the *trans* interaction between Thy-1 and integrin, namely αvβ3, αXβ2, and αMβ2 (Rege and Hagood, 2006a). Therefore, the *trans* interaction mediates mechanotransduction in the context of cell-cell interaction, which result in either clustering of Thy-1 and subsequent suppression of c-Src (Figure 1 ①) or directly force transduction (Figure 1②). The *cis* interaction, on the other hand, plays a more inhibitive/regulatory role in integrin mediated mechanotransduction. Through direct binding to integrin through its RLD motif, Thy-1 can restrict integrin by promoting its bent conformation, due to the proximity of the RLD motif against the plasma membrane. Furthermore, through interaction with integrin, Thy-1 can also effectively reduce overall integrin avidity toward ECM ligands (Figure 1③a) and at the same time, inhibit integrin-mediated latent TGF-β activation (Figure 1④).

The impacts of Thy-1 on cytoplasmic mechanotransduction pathway, on the other hand, are indirect due to the lack of a Thy-1 cytoplasmic domain. In addition to regulating integrin affinity/avidity through direct *cis*-interaction, Thy-1-integrin binding also facilitates phosphorylation and recruitment of CBP through Fyn, a lipid raft Src family kinase (SFK) recruited to the focal adhesion by Thy-1. Subsequently, Csk is recruited by CBP, leading to phosphorylate the c-terminus of and inactivate c-Src. The c-Src inhibition subsequently leads to reduced p^190^GAP activity and elevated RhoA-dependent actin stress fiber assembly. It has been widely described that RhoA/ROCK controlled cellular G-actin pool dynamics directly regulates nuclear translocation and activation of MRTF (Miralles et al., 2003; Fan et al., 2007; Vartiainen et al., 2007). Therefore, Thy-1 mediated downregulation of Src activity can result in nuclear accumulation of active MRTF in response to extracellular tension by reducing availability of MRTF-inhibitory G-actin (Figure 1③b). Similarly, both Hippo dependent and independent Yap/Taz signal transduction are also tightly regulated by RhoA mediated F-actin stress fiber assembly (Dupont et al., 2011; Sansores-Garcia et al., 2011; Wada et al., 2011). Besides a relatively “slower” pathway to regulate RhoA activity through Src, Fyn has also been shown to directly activate Rho GEF LARG and thus enables swift early cellular response toward extracellular mechanic cues through RhoA. In sum, through indirectly manipulating RhoA activity and subsequent equilibrium between G-actin and F-actin, Thy-1 functions as a key regulator of cellular mechanotransduction.

The impact of Thy-1 on mechanotransduction is likely the fundamental mechanism behind its broad functionality. This mechano-based regulatory mechanism not only affects cellular behavior but also profoundly influence tissue development and cell differentiation. Thy-1 negative fibroblasts are more sensitive to inflammatory cytokines and more likely to differentiate into myofibroblasts (Sanders et al., 2007). Thy-1 deficiency also leads to poor osteogenesis in mouse due to altered Wnt pathway (Picke et al., 2018). Without Thy-1, mouse mesenchymal stem cells (MSC) are more likely to differentiate into adipocytes instead of osteoblasts (Picke et al., 2018). These discoveries strongly suggest that Thy-1, through integrin mediated mechanotransduction, significantly influences differentiation and cell fate determination. Recently it has been reported that integrin αvβ3 signaling potentiates fibrotic activation of lung fibroblast (Fiore et al., 2018). In the study, Thy-1 KD induced fibroblast stiffening and promoted MRTF nucleus translocation with enhanced cellular contractility. Elevated αvβ3 staining was observed in both Thy-1 KD cells and in Thy-1 null mice treated with bleomycin to induce lung fibrosis, implicating strong correlation between lung fibrogenesis, Thy-1 loss and dysregulated integrin αvβ3 signaling.

Unlike other mechanotransducing molecules, Thy-1 is capable of mediating mechanotransduction through *trans* AND *cis* interactions with integrins, making the GPI anchored protein a unique mechano mediator. The Thy-1 mediated mechanotransduction is highly context dependent. The *trans* molecular coupling of Thy-1 with integrin and Syndecan4 is necessary to generate full strength of force as well as cellular contractile formation. Meanwhile, the lipid raft GPI anchor is an absolute requirement for Thy-1 *cis* mechanotransduction, emphasizing the importance of lipid environment for proper Thy-1 functionality. Considering that the function of Thy-1 is also highly cell type dependent, it is conceivable that differential membrane protein coupling and subtle change in lipid raft composition could serve as a fine-tuned regulatory mechanism of Thy-1 mediated mechanotransduction. Therefore, Thy-1 could potentially be coupling with other not-yet-identified lipid raft proteins directly or indirectly and thus regulating a wide range of mechano-related cellular response spanning from ECM remodeling to cell differentiation and determination. More studies are needed to fully understand the role of Thy-1 in the context of mechanotransduction.

## Author Contributions

PH and TB conceived the idea and wrote the manuscript. TB conceptualized and created the figure.

### Conflict of Interest Statement

The authors declare that the research was conducted in the absence of any commercial or financial relationships that could be construed as a potential conflict of interest.
